# P-1271. Occurrence of Avian Pathogenic *Escherichia coli* (APEC) with potential for human extraintestinal infections in poultry environments of Bangladesh

**DOI:** 10.1093/ofid/ofae631.1452

**Published:** 2025-01-29

**Authors:** Md Badrul Amin, Kazi Injamamul Hoque, Ajrin Sultana Sraboni, Md Omar Bhuiyan, Rubaiya Jannat Poroma, Mohammad Aminul Islam

**Affiliations:** icddr,b, Dhaka, Dhaka, Bangladesh; icddr,b, Dhaka, Dhaka, Bangladesh; icddr,b, Dhaka, Dhaka, Bangladesh; icddr,b, Dhaka, Dhaka, Bangladesh; icddr,b, Dhaka, Dhaka, Bangladesh; Washington State University, Pullman, Washington

## Abstract

**Background:**

Avian pathogenic *E. coli* (APEC) causes colibacillosis mainly in poultry industry. Previous reports suggest that APEC strains often harbor virulence factors that are related to *E. coli* causing extraintestinal infections in humans known as ExPEC, highlighting their zoonotic potential. However, limited information is available on APEC harboring ExPEC specific genes in Bangladesh. This study aimed to isolate and characterize APEC strains carrying ExPEC genes in urban and rural poultry environments.

Prevalence of of Avian pathogenic E. coli in different poultry samples collected from urban poultry markets and rural poultry farms. LBM, urban live bird market; RPF, rural poultry farm; GIT, gastrointestinal tract

**Figure:**
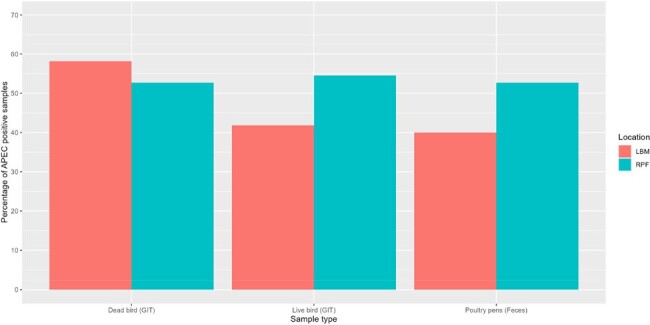

**Methods:**

During 2019–21, we collected 330 gastrointestinal tracts (GIT) of freshly slaughtered poultry from urban live bird markets (LBM) (n=55) and rural poultry farms (RPF) (n=55), GIT of dead poultry from LBM (n=55) and RPF (n=55), along with feces from poultry pens of LBM (n=55) and RPF (n=55). We tested these samples for *E. coli* by direct plating on TBX agar. Biochemically confirmed five *E. coli* colonies per sample were investigated for both APEC (*iroN, ompT, hlyF, iss, iutA*) and ExPEC (*cvaA/B5/C, etsA/B, kpsMTK1, kpsMTII, sfsS, ompA, ace35*) associated genes using PCR. All the APEC isolates were tested for antibiotic sensitivity to 21 antibiotics, phylogrouping and a subgroup of APEC strains carrying ExPEC genes was subjected to whole genome sequencing.

Phylogenetic analysis of APEC strains carrying ExPEC associated virulence genes. MLST, multi locus sequence type.

**Figure:**
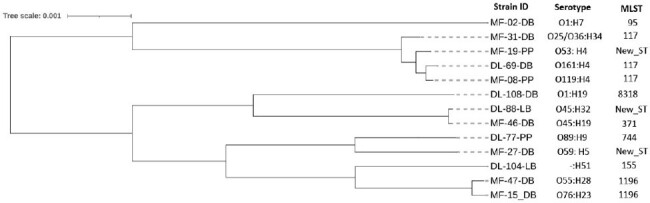

**Results:**

All 330 samples were *E. coli* positive with a mean count of 6.4 (SD 1.5) log_10_ CFU/g of GIT. Around 50% of samples (n=165) were APEC positive with similar trend of prevelance in both LBM (53%, n=88) and RPF (47%, n=77) (Fig. 1). All APEC isolates were multi drug resistant (MDR), with highest resistance to macrolides (100%), penicillins (98%), tetracyclines (96%), fluoroquinolones (93%), folate inhibitors (89%), phenicols (53%), aminoglycosides (33%), cephalosporins (13%) and carbapenems (4%). Predominant phylogroups were B1 (10%) followed by F (7%), E (5%) and A (4%). Around 68% of APEC isolates (n=113) harbored ExPEC associated genes and were genetically diverse in terms of their serotypes, sequence types except ST117 and 1196, and single nucleotide polimorphism (Fig. 2).

**Conclusion:**

The higher prevalence of MDR APEC with ExPEC traits underlines the risk of human infection given the zoonotic transmission of the organisms. Public health measures should be taken to control the potential spillover of the organisms from poultry to humans.

**Disclosures:**

**All Authors**: No reported disclosures

